# Construction of Binary RGO/TiO_2_ Fibrous Membranes with Enhanced Mechanical Properties for *E. coli* Inactivation

**DOI:** 10.3390/nano13222954

**Published:** 2023-11-15

**Authors:** Suyi Zhao, Zhenzeng Chong, Xiaogang Zuo, Wenjun Qi

**Affiliations:** 1 Materials Science and Technology, Xinjiang University, Urumchi 830046, China; 107551900933@stu.xju.edu.cn; 2 Materials Science and Technology, Dongnan University, Nanjing 211189, China; 230238713@seu.edu.cn; 3 Aluminum-Based Industrial Innovation Research Institute of Xinjiang, Urumchi 830013, China; 107552200723@stu.xju.edu.cn

**Keywords:** RGO/TiO_2_, nanomembrane, photocatalytic, sterilization

## Abstract

For environmental remediation, it is significant to design membranes with good mechanical properties and excellent photocatalytic activity. In this work, RGO/TiO_2_ membranes with heterogeneous structures and good photocatalytic efficiency were synthesized using the method of electrospinning combined with a thermal treatment process. In the binary nanocomposites, RGO was tightly adhered to TiO_2_ fibers and by simply adjusting the loading of RGO, the strength and modulus of the fibrous membranes were improved. Notably, the RGO-permeated TiO_2_ fibers exhibited 1.41 MPa in tensile strength and 140.02 MPa in Young’s modulus, which were 705% and 343% of the original TiO_2_ fibers, respectively. Benefiting from the enhanced light response and the homogeneous and compact heterogeneous structure, the synthesized RGO/TiO_2_ membranes displayed good antibacterial performance with a photocatalytic inactivation rate of 6 log against *E. coli* within 60 min. This study offers a highly efficient alternative to inactivate *E. coli* for the synthesis of TiO_2_-based membranes.

## 1. Introduction

As industrialization continues to develop, there has been a gradual increase in water pollution, which has become a serious threat to the balance of the ecosystem and to human health. Due to their low degradability, antibiotics and pathogenic bacteria are major pollutants in wastewater [1,2,3]. Thus, the efficient removal of antibiotics and pathogenic bacteria from wastewater has become a hot topic in current environmental research. Although several disinfection techniques have been developed for microbial inactivation, such as chlorination, UV irradiation, physical and chemical adsorption, it is inevitable that they produce harmful sterilization by-products [4,5]. Photocatalytic oxidation technology has shown tremendous potential for its application in water disinfection with respect to the conventional methods of bacterial inactivation. Among various semiconductor photocatalysts, titanium dioxide (TiO_2_) is highly recognized in terms of its unique properties, which include high chemical inertness, low cost, and excellent photo-chemical performance [6]. However, the relatively large band gap (3.2 eV) of TiO_2,_ which limits its absorption of visible light, and the low mechanical strength of TiO_2_ fibers, mainly in the form of fracture, present the problem that the catalyst cannot be easily separated and recovered in liquid-phase photocatalytic reactions [7,8]. To solve this problem, various methods have been developed to enhance TiO_2_ fibers, including making TiO_2_ with a high specific area and three-dimensional open structure, infiltrating polymers into TiO_2_ fibers, and constructing heterojunctions by coupling narrow band gap semiconductors to enhance the photocatalytic performance of the material [9,10,11,12].

Reduced graphene oxide (RGO) is a two-dimensional carbonaceous material with unique properties, such as high carrier mobility, high thermal conductivity, high specific surface area and transparency. Owing to the fact that the Fermi level of RGO is lower than the conduction band (CB) edge of TiO_2_, the photo-generated electrons in TiO_2_ can be easily transferred to dioxygen or protons through the RGO sheets, thus facilitating charge-pair separation [13]. In addition, reduced graphene oxide (RGO) possesses abundant carboxyl and hydroxyl functional groups on the surface and edges, which can increase the active adsorption sites through electrostatic and hydrogen bonding, and p-p, n-p, and van der Waals interactions, thus enhancing the adsorption capacity of the photocatalysts for a variety of pollutants. Thus, RGO has attracted much attention in the field of visible photocatalysis [14,15].

Recently, the construction of heterostructures by introducing RGO coupled with wide band gap n-type semiconductor TiO_2_ has become a new research hotspot [16,17]. For example, photocatalysts of TiO_2_ nanoparticles modified via RGO under visible-light-induced conditions, and the hydrophilicity of organic pollutants during the photocatalytic reaction and their antibacterial properties, were significantly enhanced [18,19]. Compared to suspended nanoparticles, electrospun nanofibrous membranes are significantly easier to fabricate. In terms of environmental remediation, the electrospun nanofibrous materials have some attractive characteristics, such as a large fiber aspect ratio and good spatial connectivity, which offer tremendous advantages for the synthesis of semiconductor nanomaterials with high reactivity and large area heterostructures on the surface of the fibers [20]. However, in the synthesized RGO/TiO_2_ nanomaterials, RGO tend to agglomerate on the surface of TiO_2_ fibers, resulting in insufficiently compact interfacial contact between RGO and TiO_2_, which is not favorable for the effective transfer of photogenerated charge carriers. In addition, some synthesized RGO/TiO_2_ membranes are particularly fragile and could easily and rapidly turn into scattered fractions during the photocatalytic process, resulting in dispersion in the water. As they are difficult to be isolated and recycled, this can cause secondary water pollution in practical applications [20,21]. Therefore, it would be of great significance to design and synthesize novel heterostructure RGO/TiO_2_ composites that not only exhibit photocatalytic disinfection but also possess good structural stability.

In this work, we propose an interesting strategy for synthesizing RGO/TiO_2_ nanofiber membranes with heterogeneous structures. In this binary membrane photocatalyst, RGO was homogeneously dispersed in TiO_2_ nanofibers to obtain composite fiber membranes of 1.41 MPa in tensile strength and 140.02 MPa in Young’s modulus. RGO/TiO_2_ fiber membranes were first prepared using an electrostatic spinning technique, and then the fiber membranes were calcined. Significantly, the as-fabricated binary RGO/TiO_2_ fiber membranes with heterogeneous structure have the advantages of good mechanical properties and enhanced visible light absorption, as well as good antibacterial properties, thus exhibiting great prospects for their application in the field of wastewater purification.

## 2. Materials and Methods

### 2.1. Chemicals

The titanium butoxide (C_16_H_36_O_4_Ti) was analytical grade and was purchased from the Shanghai Aladdin Biochemical Technology Co., Ltd. (Shanghai, China). Polyvinylpyrrolidone ((C_6_H_9_NO)_n_, M_W_ = 1,300,000) was analytical grade and purchased from the Shanghai McLean Biochemical Technology Co., Ltd. (Shanghai, China). Reduced graphene oxide (C_x_O_y_H_z_) was analytical grade and purchased from the Kaina Carbon New Material Co., Ltd. (Xiamen, China). Ethanol (CH_3_CH_2_OH) was analytical grade and purchased from the Tianjin Xinbute Chemical Co., Ltd. (Tianjin, China). Glacial acetic acid (CH_3_CO_2_H) was analytical grade and purchased from the Tianjin Zhiyuan Chemical Reagent Co., Ltd. (Tianjin, China). All chemicals were used as received without further purification.

### 2.2. Fabrication of RGO/TiO_2_ Nanofibrous Membranes

Normally, 1 g of PVP is dissolved in 9 g of ethanol and 8 g of acetic acid and stirred continuously for 2 h. A certain mass of reduced graphene oxide and TBT was added slowly into the prepared polymer solution (the ratios of reduced graphene oxide to the total solution mass were 0.5:1000, 1:1000, 1.5:1000, 2:1000). The obtained samples were signified as 1-RGO/TiO_2_, 2-RGO/TiO_2_, 3-RGO/TiO_2_, and 4-RGO/TiO_2_, respectively. Then, the prepared solution was stirred continuously for 30 min to obtain the black precursor with PVP as the polymer template.

**Figure 1:**
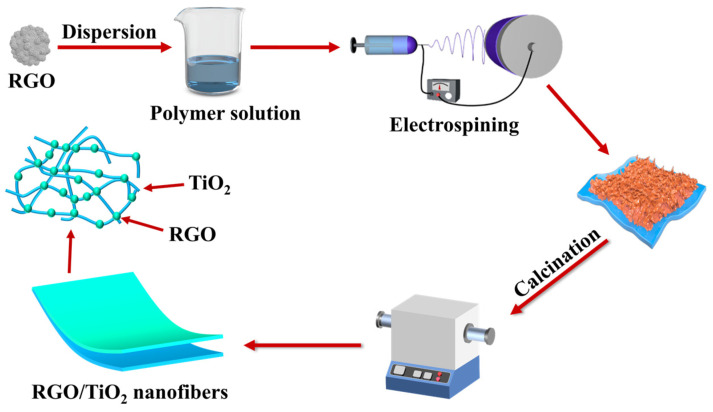
Schematic illustration for the fabrication of RGO/TiO_2_ nanofibrous membranes.

The TL-BM 300 electrostatic spinning machine was used to spin the above-mentioned prepared precursor solutions. The specific spinning process parameters were set as follows: the working voltage was 15 kV, while the feeding speed was kept at 1.5 mL/h; the distance between the spinning nozzle and the receiving drum was 15 cm, the spinning temperature environment was controlled at 25 ± 2 °C, and the relative humidity was controlled at 45 ± 2%. After the spinning process, the precursor nanofibrous membranes were placed in a vacuum drying oven and dried at 50 °C for 6 h to remove the residual solvent. Finally, the RGO/TiO_2_ membrane was transferred to a tube furnace for calcination at a rate of 5 °C/min to 550 °C and maintained for 1 h to remove the polymer template. For comparative studies, pure TiO_2_ fiber membranes were prepared using the same method described above, and the detailed pathway of RGO/TiO_2_ membranes was shown in Figure 1.

### 2.3. Characterization

The morphologies and energy-dispersive X-ray spectroscopy (EDS, Hitachi, Ltd., Tokyo, Japan) elemental mapping of the prepared membranes were observed using a Hitachi SU8010 scanning electron microscope (SEM, Hitachi, Ltd., Tokyo, Japan). Transmission electron microscopy (TEM, JEOL, Ltd., Tokyo, Japan) of the images was performed using a JEOL JEM-2100F. X-ray diffraction (XRD, Bruker, Ltd., Karlsruhe, Germany) was measured on a Bruker D8 Advance X-ray diffractometer (λ = 0.15406 nm for Cu Kα irradiation). X-ray photoelectron spectra measurement was performed on a Thermo Fisher Scientific ESCALAB250Xi (XPS, Thermo Fisher Scientific, Ltd., Waltham, MA, USA). Raman spectra analysis of as-fabricated membranes was characterized using a Horiba HR Evolution (laser wavelength: 633 nm, power: 0.51~0.68 mW, resolution ≤ 1 cm^−1^, Horiba, Ltd., Palaiseau, French). The functional groups of membranes at 400–4000 cm^−1^ were analyzed using a Fourier infrared spectrometer of Bruker model VERTEX70 (FTIR, Bruker, Ltd., Karlsruhe, Germany) with a signal resolution of 2.0 cm^−1^. UV-visible diffuse reflection spectrum (DRS) was measured for various photocatalysts employing a spectrophotometer (Hitachi UV3600, Shimadzu, Ltd., Kyoto, Japan). Measurements of the tensile strength of the fiber membranes were carried out using an electronic universal biomaterials tensile tester in accordance with ISO 1798:2008 standard (scale 3 × 0.5 cm^2^, thickness 50 ± 5 μm, upper and lower fixture spacing 1 cm, and stretch speed at 5 mm/min^−1^).

### 2.4. Antibacterial Experiments

After incubation of Gram-negative *E. coli* in fresh nutrient broth for 24 h at 37 °C, the membrane was centrifuged and washed three times with phosphate-buffered saline, which was then diluted to obtain an initial bacterial concentration of approximately 2 × 10^9^ CFU mL^−1^. The 2 mg (size of 1 × 1 cm^2^) membrane was immersed in 10 mL of bacterial suspension (pH value of 7) and then photocatalytically sterilized using a 300 W Xe/UV lamp. Bacterial suspensions, which were sampled and continuously diluted at each time point, were then laid on nutrient broth agar to conduct bacterial counts. For comparison, antimicrobial experiments were performed on pure TiO_2_, 1-RGO/TiO_2_, 2-RGO/TiO_2_, 3-RGO/TiO_2_, and 4-RGO/TiO_2_ membranes under UV and visible-light irradiation. There were three duplicates of antimicrobial experiments, all of which were measured under sterilized conditions.

## 3. Results and Discussion

### 3.1. Morphology and Structures of RGO/TiO_2_ Nanofibrous Membranes

**Figure 2:**
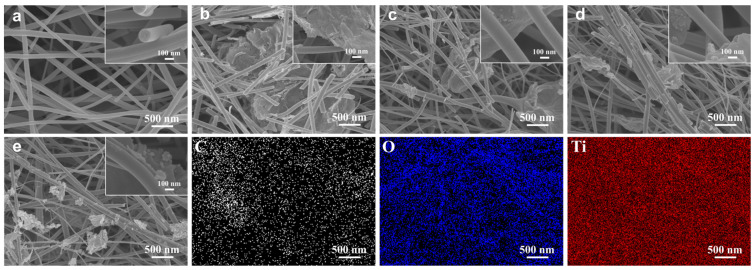
SEM images of (**a**) TiO_2_, (**b**) 1-RGO/TiO_2_, (**c**) 2-RGO/TiO_2_, (**d**) 3-RGO/TiO_2_, (**e**) SEM image of 4-RGO/TiO_2_ with elemental mapping images of C, O, and Ti, respectively.

Initially, the surface morphology of the manufactured membranes was characterized through SEM analysis. Figure 2a shows the electrospun TiO_2_ nanofiber material consisting of the randomly oriented nanofibers, which had smooth fiber surfaces and an average fiber of approximately 248 nm in diameter. Moreover, the three-dimensional open pore mesh structure formed by cross-stacking between fibers was beneficial to the growth of RGO on the fiber surface. It could be seen from the SEM that after the initial doping of reduced graphene oxide, more broken fibers appeared in TiO_2_, which may be due to the decrease in the conductivity of the polymer solution as a result of RGO doping. With the increase in RGO doping, TiO_2_ fractured fibers started to decrease, the fine RGO nanoparticles were tightly and firmly adhered to the TiO_2_ nanofibers, and the agglomerate-shaped RGO coated the TiO_2_ nanofibers to support the fiber backbone structure (Figure 2b,c). When the RGO doping amount reached 1.5:1000, an obvious adhesion structure appeared between the TiO_2_ nanofibers, and the average diameter of the blended fibers gradually increased, which could be attributed to the fact that the increase in the RGO content affected the viscosity of the spinning solution (Figure 2c). When the RGO doping increased further, the fiber diameter began to decrease, the fine RGO nanoparticles adhered to the fiber surface and dispersed among the fibers, and the TiO_2_ fiber film was able to form a dense mesh structure (Figure 2d). From the elemental spectrum of the 4-RGO/TiO_2_ fiber membrane, it could be seen that three elements, C, O and Ti, were observed in the sample, and were uniformly dispersed inside the fiber membrane.

**Figure 3:**
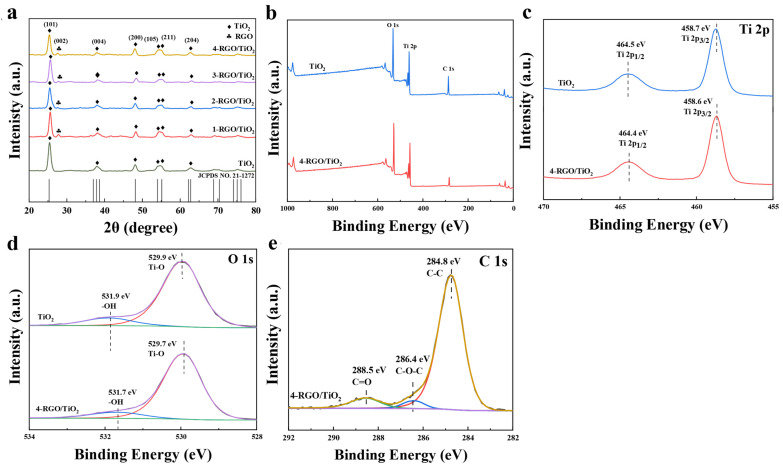
(**a**) XRD spectra of TiO_2_, 1-RGO/TiO_2_, 2-RGO/TiO_2_, 3-RGO/TiO_2_, 4-RGO/TiO_2_; (**b**) XPS spectra of TiO_2_ and 4-RGO/TiO_2_ fibrous membranes, (**c**) Ti 2p, (**d**) O 1 s, (**e**) C 1 s.

Figure 3a shows the XRD diffractograms of the prepared TiO_2_, 1-RGO/TiO_2_, 2-RGO/TiO_2_, 3-RGO/TiO_2_, and 4-RGO/TiO_2_ nanofibrous membranes. Diffraction peaks at 2θ of 25.2°, 37.4°, 47.8°, 53.3°, and 62.1° for TiO_2_ fibrous membranes correspond to the anatase phase of (101), (004), (200), (105), (211), and (204) crystal planes, respectively [22]. Compared with TiO_2_, the diffraction peak was exhibited at 2θ of 26.5° for the RGO/TiO_2_ nanofibrous membranes, in accordance with the (002) crystal plane of reduced graphene oxide, indicating that the RGO was well doped into the TiO_2_ nanofibers [23,24]. In addition, XPS analysis was also performed to characterize the chemical state of the manufactured TiO_2_, 4-RGO/TiO_2_. In Figure 3b, the measured spectra revealed the presence of Ti, O and C, which was consistent with the results of previous EDS analyses. And the signal of O 1 s originated from the hydroxyl group (-OH) on the surface of the sample [25]. As for the pristine TiO_2_, the signal of C 1 s originated from externally contaminated carbon. Figure 3c shows the high-resolution spectrum of Ti 2p; TiO_2_ showed two characteristic peaks at 464.5 and 458.7 eV, which were assigned to Ti 2p_1/2_ and Ti 2p_3/2_, respectively [26]. Compared with TiO_2_, the Ti 2p peak on 4-RGO/TiO_2_ appeared significantly blue-shifted, indicating that some of the electrons on RGO were transferred to TiO_2_ through strong interfacial interactions [27]. As depicted in Figure 3d, the two O 1 s peaks at 529.7 eV and 531.7 eV for RGO/TiO_2_ originated from the surface hydroxyl (-OH) and lattice oxygen (Ti-O), respectively [28]. The C 1 s spectrum of 4-RGO/TiO_2_ (Figure 3e) could be divided into three major peaks at 284.8, 286.4, and 288.5 eV, which were attributed to the zero-valent carbon (C-C), epoxy (C-O-C), and C-OOH groups in the RGO/TiO_2_ heterojunction, respectively [29].

**Figure 4:**
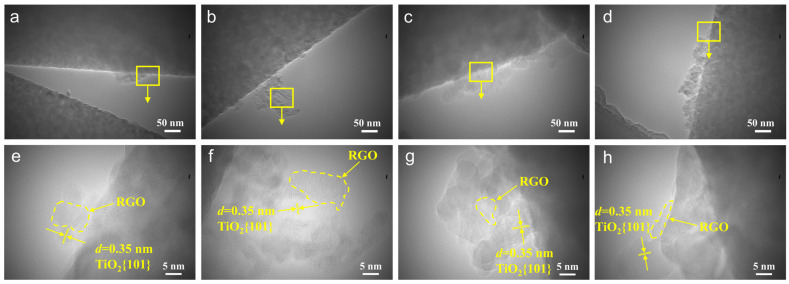
(**a**–**d**) TEM and (**e**–**h**) HRTEM images of 1-RGO/TiO_2_, 2-RGO/TiO_2_, 3-RGO/TiO_2_, and 4-RGO/TiO_2_ fibers.

To further investigate the microstructure of the nanofiber membrane, the samples were observed through TEM and HRTEM. After RGO doping, as shown in Figure 4a,e, the 1-RGO/TiO_2_ nanofibers consisted of many small anatase TiO_2_ grains and RGO, and the RGO nanoparticles were tightly and firmly adhered to the smooth fiber surface, which was consistent with the results of previous SEM and XRD analysis. As depicted in Figure 4b,f, RGO was tightly adhered between the TiO_2_ fibers and firmly adhered to the TiO_2_ nanofibers, forming heterojunctions with the TiO_2_ nanoparticles [30]. With the increase in RGO doping, the TiO_2_ nanoparticles were tightly encapsulated by the RGO nanosheet layer, and the contact interface between RGO and TiO_2_ nanofibers acted as a lattice excess, forming many miniature p-n heterojunctions (Figure 4c,g) [31]. Furthermore, the observed lattice stripes in both 3-RGO/TiO_2_ and 4-RGO/TiO_2_ fibers demonstrated the well-defined crystallinity of the fibrous membranes, where the lattice stripe spacing of 0.35 nm was in accordance with the (101) crystal plane of TiO_2_ [32]. Interestingly, with the further increase in doping, 4-RGO/TiO_2_ grew a two-dimensional lamellar structure on the fibers surface, forming a backbone structure to support the TiO_2_ nanofibers and increasing the specific surface area of TiO_2_-based nanofibers during the photocatalytic reaction (Figure 4d,h) [33].

### 3.2. Mechanical Properties of RGO/TiO_2_ Nanofibrous Membrane

**Figure 5:**
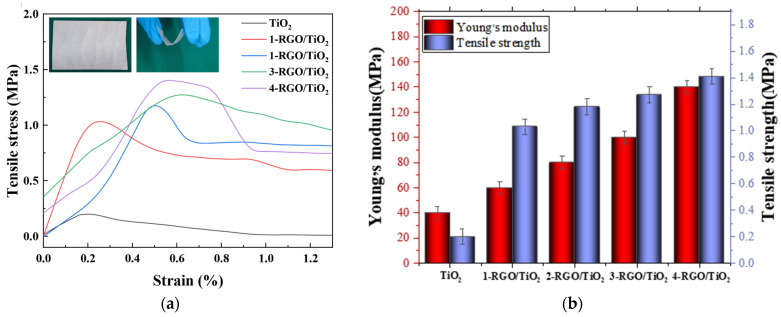
(**a**) Tensile stress–strain curves and (**b**) Young’s modulus and bending rigidity of TiO_2_, 1-RGO/TiO_2_, 2-RGO/TiO_2_, 3-RGO/TiO_2_, and 4-RGO/TiO_2_ membranes.

The mechanical properties of nanofiber membranes are critical for their use in practical photocatalytic applications. Figure 5a shows the stress–strain curves of TiO_2_, 1-RGO/TiO_2_, 2-RGO/TiO_2_, 3-RGO/TiO_2_, and 4-RGO/TiO_2_ nanofibrous membranes. The tensile strength of pristine TiO_2_ fibrous membranes was 0.2 MPa. After the modification of RGO, the tensile strength of RGO/TiO_2_ fibrous membranes showed a gradual increase, and finally, 1-RGO/TiO_2_, 2-RGO/TiO_2_, 3-RGO/TiO_2_, and 4-RGO/TiO_2_ fiber membranes reached tensile strengths of 1.03, 1.18, 1.27, and 1.41 MPa, respectively. This phenomenon was attributed to the formation of an adhesion structure between the TiO_2_ fibers. The main cause of this was that the introduced RGO nanostructures increased the friction between the fibers and the multi-level nanostructures grown between the fibers impeded the slip of the fibers during deformation, leading to an increase in the tensile strength. In addition, Young’s modulus was an important mechanical property index that reflected the stiffness of materials. As illustrated in Figure 5b, the Young’s moduli of 3-RGO/TiO_2_ and 4-RGO/TiO_2_ fibrous membranes were 100.74 and 140.02 MPa, respectively. They were higher than that of pristine TiO_2_ fiber membranes (40.72 MPa), confirming that this multistage nanostructure could enhance the mechanical properties of fibrous membranes. For RGO/TiO_2_ nanofibers, the doping of RGO played a significant role in enhancing the structural stability of TiO_2_ nanofibers, which not only inhibited the growth of grains but also reduced the defects on the fiber surface; the reduction in grain size caused the increase in grain boundaries, which was beneficial to the transfer and dissipation of stress inside the fibers [34,35]. According to the Hall–Petch theory, it could be concluded that polycrystalline nanofibers with ultra-fine grains undergo a “grain boundary slip” effect during the deformation process, which enhances the mechanical strength of nanofibers [36].

### 3.3. Optical Properties of RGO/TiO_2_ Nanofibrous Membrane

**Figure 6:**
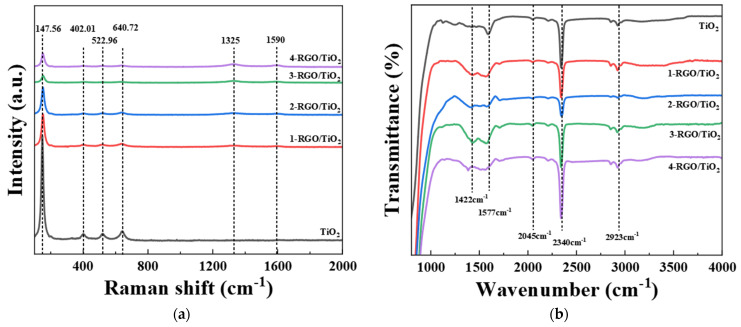
(**a**): Raman spectra analysis of TiO_2_, 1-RGO/TiO_2_, 2-RGO/TiO_2_, 3-RGO/TiO_2_, 4-RGO/TiO_2_ fiber membranes; (**b**): FTIR spectra analysis of TiO_2_, 1-RGO/TiO_2_, 2-RGO/TiO_2_, 3-RGO/TiO_2_, and 4-RGO/TiO_2_ fiber membranes.

Figure 6a shows the Raman spectra of the prepared TiO_2_, 1-RGO/TiO_2_, 2-RGO/TiO_2_, 3-RGO/TiO_2_, and 4-RGO/TiO_2_ fibrous membranes. As shown in Figure 6a, the O-Ti-O variable angle vibrational peak of the *E_g_* symmetry type in the anatase phase corresponded to the absorption peak at 147.56 cm^−1^. The absorption peak at 402.01 cm^−1^ was in accordance with the O-Ti-O variable angle vibration peak of B_1g_ symmetry type of anatase phase, with O-Ti-O symmetric bending as the main vibration and anti-symmetric stretching as the supplementary vibration. At 522.96 cm^−1^, the absorption peak corresponded to the O-Ti-O variable angle vibration peak of symmetric type A_1g_+B_1g_ of anatase phase. At 640.72 cm^−1^, the absorption peak was in accordance with the O-Ti-O variable angle vibration peak. As depicted in Figure 6a, all RGO/TiO_2_ fibers exhibited the D peak (1325 cm^−1^) and G peak (1590 cm^−1^) of RGO, which was mainly composed of symmetric C=C covalent bonds, confirming that RGO was mixed into the TiO_2_ fibers. With the increase in RGO content, the characteristic peaks of RGO in the Raman spectra became more obvious [37].

Figure 6b exhibits the FTIR spectra of the prepared TiO_2_, 1-RGO/TiO_2_, 2-RGO/TiO_2_, 3-RGO/TiO_2_, and 4-RGO/TiO_2_ fibrous membranes. The absorption peaks in the range of 1000–1400cm^−1^ were in accordance with the stretching vibrations of oxygen-containing groups in RGO, including hydroxyl (C-O, 1055 cm^−1^), epoxy (C-O-C, 1200 cm^−1^), and (C-OH, 1372 cm^−1^), respectively [38]. The characteristic peak centered at 1422 cm^−1^ corresponded to the tensile and bending vibrations of Ti-O-C in TiO_2_ fibers. The characteristic peak centered at 1577 cm^−1^ was ascribed to the C=C stretching vibration on the benzenoid ring-like structure in RGO [39]. The characteristic peak centered at 2340 cm^−1^ and might be caused by the physically adsorbed H_2_O molecules in RGO/TiO_2_ fiber membranes and atmospheric adsorbed CO_2_ molecules [40]. The characteristic peak centered at 2923 cm^−1^ originated from the stretching vibration of C-H in RGO. The characteristic peak centered at 3303 cm^−1^ was in accordance with the stretching and bending vibrations of the hydroxyl (OH^−^) group in RGO/TiO_2_ fibers.

### 3.4. Photocatalytic Antibacterial Performance of RGO/TiO_2_ Nanofibrous Membrane

**Figure 7:**
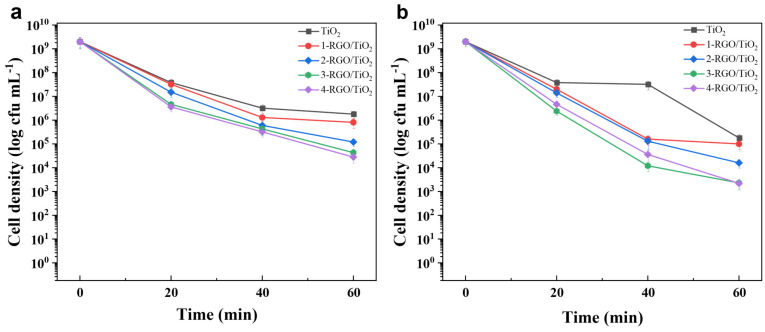

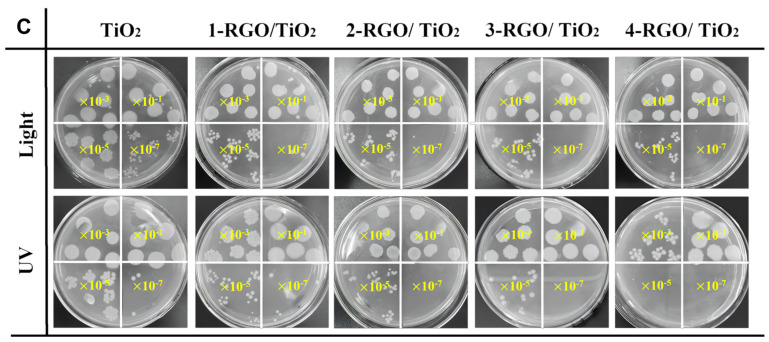
Disinfection efficiency against *E. coli* over TiO_2_, 1-RGO/TiO_2_, 2-RGO/TiO_2_, 3-RGO/TiO_2_, 4-RGO/TiO_2_ nanofibrous membranes. (**a**) under visible-light irradiation and (**b**) under UV-light irradiation. (**c**) Photographs of *E. coli* colonies treated with different photocatalysts under UV-light irradiation and visible light irradiation for 60 min, respectively.

To evaluate the photocatalytic antimicrobial performance of RGO/TiO_2_ fiber membranes, one of the typical waterborne bacteria, *E. coli*, was chosen as a model bacterium. In Figure 7a, the TiO_2_ photocatalyst had a bactericidal effect on *E. coli* under light conditions due to the generation of reactive hydroxyl groups, such as (·OH, ·O_2_^−^, H_2_O_2_, and h^+^), on the fiber surface during the photocatalytic reaction of TiO_2_ fibers that could attack the bacterial cells and cause bacterial inactivation with bactericidal effect [41]. For samples TiO_2_, 1-RGO/TiO_2_, 2-RGO/TiO_2_, 3-RGO/TiO_2_, and 4-RGO/TiO_2_ fiber membranes, 3 log, 3.3 log, 4.2 log, 4.7 log, and 4.9 log of *E. coli* were inactivated after 60 min of visible light irradiation, respectively, indicating their cytotoxic effect on *E. coli* cells. In Figure 7b, for TiO_2_ fibrous membranes after 60 min irradiation under visible light conditions, 4 log of E.coli were inactivated. The inactivation rates of 1-RGO/TiO_2_, 2-RGO/TiO_2_, 3-RGO/TiO_2_, and 4-RGO/TiO_2_ fiber membranes were 4.2 log, 5.1 log, 5.1 log, and 6 log for E.coli under the same conditions, respectively, and the 4-RGO/TiO_2_ fiber membranes exhibited excellent photocatalytic antibacterial performance.

### 3.5. Photocatalytic Mechanism Analysis

**Figure 8:**
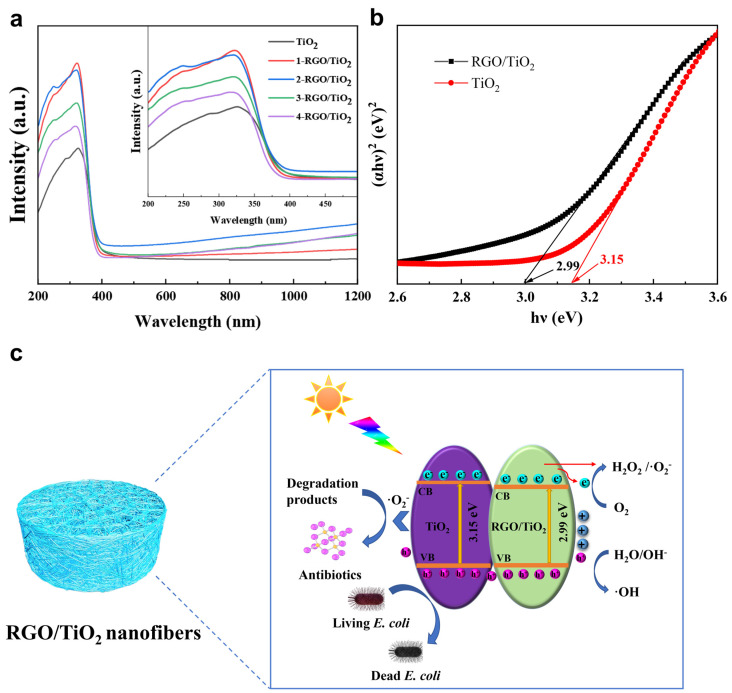
(**a**) UV–vis diffuse reflectance spectra of the TiO_2_, 1-RGO/TiO_2_, 2-RGO/TiO_2_, 3-RGO/TiO_2_, 4-RGO/TiO_2_ nanofibrous membranes; (**b**) band gaps of the TiO_2_, 4-RGO/TiO_2_ nanofibrous membranes; (**c**) proposed photocatalytic mechanism of the RGO/TiO_2_ fibrous membranes.

Figure 8a displays the UV-vis diffuse reflectance spectra of TiO_2_, 1-RGO/TiO_2_, 2-RGO/TiO_2_, 3-RGO/TiO_2_, and 4-RGO/TiO_2_ photocatalysts. The pure TiO_2_ fiber membrane merely had a response to UV light (λ < 400 nm) because of the large intrinsic forbidden bandwidth of the anatase phase TiO_2_ photocatalyst. Compared with the pure TiO_2_ fiber membrane, the absorption edge of the RGO/TiO_2_ fiber membrane gradually extended to visible light, which resulted from the RGO doping. Notably, with the increase in RGO doping, the visible-light response of RGO/TiO_2_ fiber membrane was significantly enhanced. This effect was attributed to the replacement of O atoms in TiO_2_ by C atoms in RGO, the widening of the valence band and narrowing of the band gap of TiO_2_, resulting in the blue-shifted absorption spectrum and easier generation of highly reactive electrons and holes on the fiber membrane surface. It is obvious that the enhanced light absorption is a crucial factor in enhancing photocatalytic activity [42].

In addition, the band gap of the synthesized photocatalyst can be determined by the following equation [43]:

$$\alpha hv=A{\left(hv-E_{g}\right)}^{n/2}$$

where *α* is the absorption coefficient, *v* is the frequency of light, *A* is a constant, and *E_g_* is the band gap energy. Meanwhile, *n* is equal to 4 for TiO_2_ fiber [44]. The calculated band gap energy for RGO/TiO_2_ fibers was 2.99 eV (Figure 8b), smaller than that of pure TiO_2_ fibers (3.15 eV), which may be ascribed to the doping effect of C atoms in the electronic structure of titanium dioxide [45].

According to the above experimental results, the photocatalytic reaction mechanism of RGO/TiO_2_ fiber membrane was proposed preliminarily. As depicted in Figure 8c, the flexible fiber membrane photocatalyst could offer an ideal reaction platform for *E. coli* inactivation in terms of its interconnected porous structure and abundant surface-active sites. Moreover, photogenerated holes in the TiO_2_ could directly react with H_2_O reaction to generate ·OH, while the photogenerated electrons on fibers could directly reduce dissolved oxygen to generate ·O_2_^−^, and h^+^ sites which act as the main active species in the reaction system, and the heterojunction on the fiber surface and inside could effectively promote the separation and transfer of photogenerated electron holes [46]. However, it is necessary to acknowledge the limitations of this work. Because this study aimed to explore the mechanism of RGO/TiO_2_ membranes in the photocatalytic inactivation, we had access to limited methods, which may affect the recycling performance of the RGO/TiO_2_ membranes in the antibacterial process. In our future work, we intend to investigate the balance between permeation and removal efficiency, as well as the filtration-enhanced photocatalytic degradation performance of the TiO_2_-based fibrous membranes.

## 4. Conclusions

In summary, using electrostatic spinning technology in combination with the impregnation process, novel heterojunction RGO/TiO_2_ nanofiber membranes were successfully fabricated. Taking advantage of the porous structure, homogenous and compact engagement interfaces among RGO nanoparticles and TiO_2_ nanofibers, as well as the effective photo-induced charge transfer, the optimal RGO/TiO_2_ nanofibrous membranes displayed outstanding performance in light-driven photocatalysis for bacterial inactivation. Moreover, the 4-RGO/TiO_2_ nanofiber membranes possessed good structural integrity, as well as enhanced mechanical properties which were attributed to the adhesion structure and the formation of binding sites between the fibers, in addition to the fact that the membranes could be directly picked out from the solution without cumbersome subsequent processing. This work not only demonstrates the feasibility of RGO/TiO_2_ fiber membranes for inactivation of *E. coli* as promising photocatalysts, but also offers new insights into the design and fabrication of functional membranes that could be flexibly applied to environmental remediation.

## Data Availability

The data that support the findings of this study are available from the corresponding author upon reasonable request.
